# Vector Specificity of Arbovirus Transmission

**DOI:** 10.3389/fmicb.2021.773211

**Published:** 2021-12-09

**Authors:** Marine Viglietta, Rachel Bellone, Adrien Albert Blisnick, Anna-Bella Failloux

**Affiliations:** Unit of Arboviruses and Insect Vectors, Institut Pasteur, Sorbonne Université, Paris, France

**Keywords:** mosquito, tick, arbovirus, vectorial transmission, host–pathogen interactions

## Abstract

More than 25% of human infectious diseases are vector-borne diseases (VBDs). These diseases, caused by pathogens shared between animals and humans, are a growing threat to global health with more than 2.5 million annual deaths. Mosquitoes and ticks are the main vectors of arboviruses including flaviviruses, which greatly affect humans. However, all tick or mosquito species are not able to transmit all viruses, suggesting important molecular mechanisms regulating viral infection, dissemination, and transmission by vectors. Despite the large distribution of arthropods (mosquitoes and ticks) and arboviruses, only a few pairings of arthropods (family, genus, and population) and viruses (family, genus, and genotype) successfully transmit. Here, we review the factors that might limit pathogen transmission: internal (vector genetics, immune responses, microbiome including insect-specific viruses, and coinfections) and external, either biotic (adult and larvae nutrition) or abiotic (temperature, chemicals, and altitude). This review will demonstrate the dynamic nature and complexity of virus–vector interactions to help in designing appropriate practices in surveillance and prevention to reduce VBD threats.

## Introduction

Vector-borne diseases (VBDs) represent almost one-fourth of annual deaths attributed to infectious diseases (Jones et al., 2008). In recent decades, growing trade and increased international tourism, have highly contributed to the expansion of vectors colonizing new territories and thus threatening new regions with new pathogens (Esser et al., 2019). These changes imply that endemic pathogens can be transmitted by imported vectors, or newly introduced pathogens can be transmitted by local vector populations.

**FIGURE 1:**
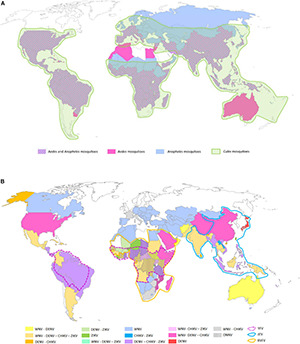
Global distributions of mosquito genera of medical importance **(A)** and arboviruses transmitted by the three main mosquito genera **(B)**. **(A)** The three mosquito genera reported on the map are the most prevalent ones around the world representing the principal vectors of arboviruses (*Aedes* and *Culex* spp.) and parasites (*Anopheles* spp.) of human health importance. The pink, blue, and green areas represent respective presence of *Aedes* spp., *Anopheles* spp., and *Culex* spp. mosquitoes. The hatched areas represent the distribution of *Aedes* spp. and *Anopheles* spp. mosquitoes in the same countries. **(B)** Map of the main arboviruses transmitted by *Aedes*, *Culex*, and *Anopheles* spp. including the flaviviruses (YFV, JEV, DENV, ZIKV, and WNV), the alphaviruses chikungunya (CHIKV) and O’nyong’nyong virus (ONNV), and the phlebovirus Rift valley fever virus (RVFV) (Tiwari et al., 2012; Hanley et al., 2013; Reisen, 2013; Fredericks and Fernandez-Sesma, 2014; Houe et al., 2019; Noorbakhsh et al., 2019; Pezzi et al., 2019; Centers for Disease Control and Prevention, 2020a,c; European Centre for Disease Prevention and Control, 2021). The maps were built using the open source map site https://cmap.comersis.com/cartes-Monde-WORLD.html.

**FIGURE 2:**
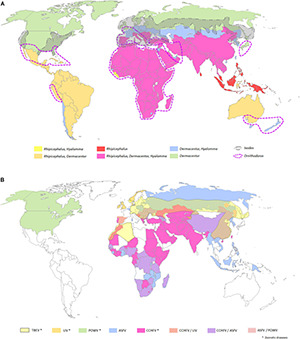
Global distributions of tick genera of medical and veterinary importance **(A)** and arboviruses transmitted by ticks **(B)**. **(A)** The five tick genera reported on the map, namely, *Ornithodoros*, *Ixodes*, *Rhipicephalus*, *Dermacentor*, and *Hyalomma* spp., are the most prevalent ones around the world representing the principal vectors of arboviruses. **(B)** Map of the main arboviruses transmitted by ticks. The yellow, orange, green, blue, and pink areas represent respective presence of the tick-borne flaviviruses, namely, tick-borne encephalitis virus (TBEV), Louping ill virus (LIV), and Powasan virus (POWV); the Asfivirus, namely, African swine fever virus (ASFV); and the Orthonairovirus, namely, Crimean-Congo hemorrhagic fever virus (CCHFV) (Myers, 2015; Dupraz et al., 2016; Diuk-Wasser et al., 2016; DW Akademie, 2016; de la Fuente et al., 2017; Frant et al., 2017; Galgani et al., 2017; Bakkes et al., 2018; Lindqvist et al., 2018; Andersen et al., 2019; Burrow et al., 2019; Kemenesi and Banyai, 2019; Wang et al., 2019; Centers for Disease Control and Prevention, 2020b; Gaudreault et al., 2020; World Health Organization, 2020). The map was built using the open source map site https://cmap.comersis.com/cartes-Monde-WORLD.html.

To be efficient, the vectorial system requires high densities of competent vectors, a high vector survival rate, and frequent contacts between vectors and susceptible vertebrate hosts. Taken together, all these parameters contribute to the vectorial capacity, which is related to the efficiency of a vector population to transmit a pathogen under natural conditions. The vectorial capacity encompasses the vector competence, which is defined as the ability of an arthropod to acquire, sustain replication and dissemination of a pathogen, and then successfully transmit it to new susceptible hosts (Monath, 1988). Differences in vector competence result from specific interactions between genetics of both vectors (vector genus, species, and population) and viruses (viral strain and genotype), which are modulated by external (biotic and abiotic) factors. Due to their worldwide distribution and their abilities to transmit various human and animal pathogens such as viruses, protozoans, bacteria, and microfilariae (Mehlhorn, 2008; Tolle, 2009), both mosquitoes (Figure 1A) and ticks (Figure 2A) are considered to be the main vectors of vector-borne pathogens (VBPs) of medical and veterinary importance. It is clear that ticks and, more particularly, the hard ticks can transmit a larger class of VBPs than mosquitoes probably due to the longer and voluminous blood meal they can absorb. While mosquitoes are not usual vectors of bacteria, ticks are typical vectors of several bacterial families such as *Anaplasmatacae*, *Francisellaceae*, *Bartonellaceae*, *Brucellaceae*, and *Spirochaetaceae* (Aubry and Geale, 2011; Stuen and Longbottom, 2011). In addition, both mosquitoes and ticks may transmit parasites; e.g., ticks transmit parasites of *Babesiiadae* family and mosquitoes transmit protozoans of *Plasmodiidae* families including *Plasmodium falciparum* that imposes a huge burden of disease. It caused around 229 million cases in 2019 in Africa, Southeast Asia, and South America. A total of 409,000 deaths were attributed to malaria in 2019, 88% of total cases being in Africa (Phillips et al., 2017).

In addition to bacteria and parasites, mosquitoes and ticks are also vectors of viruses named arboviruses (arthropod-borne viruses). Around 500 arboviruses are already described worldwide, but only some of them are pathogenic for animals and/or humans. Among them, about 50 arboviruses affect domestic animals and wildlife, and more than 100 could be pathogenic for humans (Gubler, 2001; Hubalek et al., 2014). With 390 million cases in 2019, dengue is the most prevalent VBD in the world (Hubalek et al., 2014; Huang et al., 2019). However, other arboviruses like yellow fever virus (YFV), Zika virus (ZIKV), Japanese encephalitis virus (JEV), chikungunya virus (CHIKV), or West Nile virus (WNV) also impact periodically human populations (Figure 1B). Regarding animals, alphaviruses such as eastern equine encephalitis virus (EEEV), Venezuelan equine encephalitis virus (VEEV), and western equine encephalitis virus (WEEV), mainly transmitted by *Culex* spp. and *Culiseta* spp. mosquitoes, kill horses [e.g., rate of mortality higher than 50% (Hayes et al., 1981; Foster, 2018)], while others such as Middleburg Virus (MIDV) and Sindbis Virus (SINV) affect horses, cattle, sheep, goats, and, to a lesser extent, pigs. In addition, the phlebovirus Rift Valley fever virus (RVFV), mostly transmitted by *Aedes* and *Culex* mosquitoes, is associated with a high mortality rate in young animals, causes abortions, and could also severely affect humans (Hubalek et al., 2014). Similar to mosquitoes, ticks are also able to transmit arboviruses affecting mostly animals and, to a lesser extent, humans (Figure 2B). Regarding tick-borne viruses (TBV), the African swine fever virus (ASFV, *Asfarviridae* family), and the Nairobi sheep disease virus (NSDv, *Nairoviridae* family) are undoubtedly the most pathogenic viruses for pigs and sheep with up to 100 and 90% of mortality rate, respectively (Labuda and Nuttall, 2004; Shi et al., 2018). ASFV is transmitted by the soft tick *Ornithodoros* spp. mostly in Africa and in Asia and, more secondarily, in Europe in wild boars (World Health Organization, 2020). NSDv is transmitted by *Rhipicephalus* and *Haemaphysalis* ticks and causes a lethal disease (e.g., hemorrhagic gastroenteritis) in ruminants with a mortality rate ranging from 30 to 90% (Hubalek et al., 2014). For humans, the most lethal TBVs are tick-borne encephalitis virus (TBEV far-eastern serotype) (mortality rate up to 30% Yoshii, 2019), and the Crimea Congo hemorrhagic fever virus (CCHFV) (mortality rates ranging from 3 to 40% Portillo et al., 2021). Among all classes of pathogens transmitted by mosquitoes and ticks, arboviruses represent undoubtedly the most significant threat for animal and human health over these last decades. Ten arbovirus families are described in mosquitoes and ticks as being important for human and animal health. Table 1 gives details on virus families, vectors, genome features (size, polarity), and examples of viruses with human or veterinary importance (Halbach et al., 2017; Kazimirova et al., 2017).

**TABLE 1:** List of arbovirus families transmitted by mosquitoes and ticks.

**Order**	**Family**	**Genus**	**Genome**	**Main vector**		**Major importance for**		**Examples**
				**Mosquito**	**Tick**	**Human**	**Animal**	
Unassigned	*Flaviviridae*	*Flavivirus*	ss RNA+	✓	✓	✓	✓	West Nile virus (WNV), Dengue virus (DENV), Tick-borne encephalitis virus (TBEV), Yellow fever virus (YFV)
	*Togaviridae*	*Alphavirus*	ss RNA+	✓	✕	✓	✓	Chikungunya virus (CHIKV), Eastern equine encephalitis virus (EEEV), O’nyong’nyong virus (ONNV), Sindbis virus (SINV)
Unassigned	*Reoviridae*	*Coltivirus*	ds RNA	✕	✓	✓	✓	Colorado tick fever virus (CTFV)
		*Orbivirus*	ds RNA	✓	✓	✕	✓	African horse sickness virus (AHSV)
Unassigned	*Asfarviridae*	*Asfivirus*	ds DNA	✕	✓	✕	✓	African swine fever virus (ASFV)
*Bunyavirales*	*Nairoviridae*	*Orthonairovirus*	ss RNA−	✕	✓	✓	✓	Crimean-Congo hemorrhagic fever virus (CCHFV), Nairobi sheep disease virus (NSDv)
	*Phenuiviridae*	*Phlebovirus*	ss RNA−	✕	✓	✕	✓	Rift Valley fever virus (RVFV), Lihan tick virus (LTV)
	*Peribunyaviridae*	*Orthobunyavirus*	ss RNA−	✓	✕	✕	✓	California encephalitis virus (CEV), La Crosse virus (LACV), Batai virus (BATV)
*Articulavirales*	*Orthomyxoviridae*	*Thogotovirus*	ss RNA−	✕	✓	✕	✓	Thogoto virus (THOV), Dhori virus (DHOV)
		*Quaranjavirus*	ss RNA−	✕	✓	✓	✓	Quaranfil virus (QRFV)
*Mononegavirales*	*Rhabdoviridae*	*Vesiculovirus*	ss RNA−	✓	✓	✕	✓	Malpais spring virus (MSPV)
	*Nyamiviridae*	*Nyavirus*	ss RNA−	✕	✓	✕	✓	Nyamanini nyavirus (NYMV), Midway nyavirus (MDWV)

*ssRNA−, single-stranded negative sense RNA; ssRNA+, single-stranded positive sense RNA; dsRNA, double-stranded RNA; dsDNA, double-stranded DNA.*

Arboviruses transmission by vectors is ensured *via* two main mechanisms named vertical (VT) and horizontal transmission (HT). Although VT maintains the virus from an infected female to her offspring by transovarian transmission, it is minor, and only few proportions of arboviruses pass through VT: California encephalitis virus (CEV) and La Crosse virus (LACV) in mosquitoes (Lequime et al., 2016) and TBEV (Rehacek, 1962) and ASFV (Rennie et al., 2001). The main transmission mode for arboviruses is HT, which is clearly dependent on the vector feeding mode. After ingestion of the virus by a vector during blood feeding, the virus replicates in the vector and infect all tissues including salivary glands where freshly produced virions can then be transmitted to a novel vertebrate host *via* saliva secreted during a subsequent blood meal. HT is predominant both in ticks and mosquitoes and is involved in infecting animals and humans by arboviruses.

However, it is naturally observed and experimentally demonstrated that although various vector species can bite the same hosts, only few species may become infected and then transmit the virus to another vertebrate host. For example, despite the close phylogenetic relatedness of o’nyong nyong virus (ONNV) and chikungunya virus (CHIKV), the first one can be transmitted by both *Aedes aegypti* and *Anopheles gambiae* (main vector of *Plasmodium* parasites), whereas the second is only transmitted by *Ae. aegypti* since CHIKV is unable to replicate in *An. gambiae* (Vanlandingham et al., 2005). Moreover, while some ticks are known to transmit a single arbovirus, others are more generalist, transmitting several arboviral families (Tokarz et al., 2018). Therefore, the functioning of a vectorial system is complex based on various factors, which are all interconnected.

In this review, we will discuss factors that may explain vector specificity, making transmission more an exception than a generality. First, vector internal factors including compatible genetic combinations of vector (mosquito and ticks) genotype and viral genotype will be examined as possible prerequisites to the functioning of the vectorial system. We will then explore how long-term interactions allow a kind of “tolerance” by the vector immune system to the virus. Second, other internal factors such as the microbiome and coinfecting arboviruses play a critical role in modulating the vector competence. Finally, all these internal factors are shaped by external factors described as biotic (nutrition and nature of blood) and abiotic factors (climate/temperature, exposure to chemicals, and topography).

## Role of Internal Factors in Virus Transmission by Mosquitoes and Ticks

The performance of the vectorial system results from long-term interactions between vectors and pathogens without substantial deleterious effects on vector’s fitness. In this section, internal factors that influence viral transmission by mosquitoes and ticks will be discussed.

### Origins of Vectors and Viruses

The diversification of *Culicinae* and *Anophelinae* lineages (both emerged from Africa) have been dated by molecular approaches to approximatively −226 Ma, meaning that the origin of mosquitoes was in the Jurassic Era (−200 to −145 Ma) (Borkent and Grimaldi, 2004; Reidenbach et al., 2009). Similarly, as *Nuttalliella* ticks are considered as ancestral and “live fossil” tick species, the tick origin has been estimated to be approximately −260 to −270 Ma (de la Fuente, 2003; Mans et al., 2011). Mosquitoes and ticks cohabited with the prehistorical hosts including birds, dinosaurs, and some small vertebrates for a long period. *Anophelinae* contains three main genera—*Anopheles* Meigen, *Bironella* Theobald, and *Chagasia* Cruz—whose ranking and relationships are still debated nowadays, since previous classifications, based on morphological characteristics, are not confirmed by molecular tools used in recent studies (Foster et al., 2017). To date, among the lineage of *Anophelinae*, the genus *Anopheles* counts up to 480 species, but only 40 are really considered as vectors of *Plasmodium* spp. parasites (Sinka, 2013). Moreover, *Aedes* mosquitoes represent a group with species having the most significant impact on human health. The cosmopolitan “domestic” *Ae. aegypti* derived from *Ae. aegypti formosus* mainly found in African forests, *Ae. aegypti* having left Africa 1,000 years ago to colonize the rest of the world (Soghigian et al., 2020).

Today, *Aedes* mosquitoes are known to be the more efficient vector of arboviruses. It is partly explained by mosquito genetic differences based on the natural history of *Ae. aegypti* sp. (Linné, 1862). This species originated from a sub-Saharan African sylvan ancestor that moved to West Africa late in the eighth century and then invaded the new world along with the African slave trade from the fifteenth to the seventeenth century. Then, around 1,800, this mosquito species was introduced in the Mediterranean basin and established in the port cities. From 1869, the Suez Canal facilitated commercial exchange and also participated to the large dissemination and the global invasion of *Ae. aegypti* into Asia (Smith, 1956), Australia (1887), and the South Pacific region (1904) (Powell et al., 2018). On the other hand, *Aedes albopictus* native to tropical forests of Southeast Asia, was mainly limited to Asia, India, and several islands in the Indian Ocean region, such as La Réunion (Mattingly, 1953) and the Seychelles (Metselaar et al., 1980), and in the Pacific region, the Mariana and Papua New Guinea islands, until the late 1970s (Elliott, 1980). *Ae. albopictus* took three decades to globally colonize the world, while *Ae. aegypti* took centuries to cover the tropical regions. In addition to genetic differences between populations, these two mosquito species are distinguished by the diversity of vertebrate hosts and pathogens they may transmit. Thus, the outcome of vector–host–pathogen interactions result from a long-term adaptation between those three partners of the vectorial triad. This adaptation could be measured by quantitative genomics and *via* quantitative trait loci (QTL) and transposable elements (TEs) analysis.

### Vector Genetics and Viral Transmission

It is well known that within the vectorial system, pathogen transmission results from a compatible interaction between viral genotype and vector genotype. Genotype × genotype interactions imply that phenotypic variation in vector competence is not only modulated by independent, additive effects of both vector and virus genotypes but also by a genetic component that is specific to each virus–vector combination (Lambrechts, 2010).

#### Quantitative Genomics

Quantitative trait locus (QTL) is a portion of a genome that controls the variation of a quantitative trait phenotypically measurable, such as insecticide resistance (Saavedra-Rodriguez et al., 2008) or vector competence (Bosio et al., 2000). Interbreeding *Ae. aegypti* and *Ae. aegypti formosus* generated progeny with QTLs on chromosomes 2 and 3 that affect midgut infection barrier and midgut escape barrier for DENV (Bosio et al., 2000). Other QTLs were also identified on the same chromosomes as associated with both midgut infection and dissemination of DENV in *Ae. aegypti* (Gomez-Machorro et al., 2004; Merkling et al., 2020). These results clearly suggest the importance of QTL and, more broadly, the genetic background of mosquito in the vector competence.

More recently, new-generation sequencing (NGS) techniques allowed the exploration of vector genomes: 1,380 MB for *Ae. aegypti* (Nene et al., 2007), 1,900 MB for *Ae. albopictus* (Chen et al., 2015; Dritsou et al., 2015), 579 MB for *Culex quinquefasciatus* (Arensburger et al., 2010), 278 MB for *Anopheles* (Holt et al., 2002), and 2.1 GB for *Ixodes scapularis* (Gulia-Nuss et al., 2016). The genome sizes reflect the long evolution from their common ancestor to the current large diversity of vector populations. The size differences observed in mosquito genomes could be explained by the presence of transposable elements (TEs), considered as intragenomic parasites (McLain et al., 1987). These elements could also serve as an evolutive clock, allowing to order and classify species in relation to each other (Wu and Lu, 2019). TEs are ubiquitously found in living organisms and are integrated into the host genome from where they are able to replicate independently and to move from one chromosomal location to another by transposition (Finnegan, 1992). Transposition events can occur in all arthropod cell lines and may depend on some signals such as *P* elements in *Drosophila melanogaster* (Kaufman et al., 1989). Transposons are classified in two distinct classes. The class I relies on RNA intermediates, giving the name of retrotransposons to this class, also subdivided into long terminal repeat (LTR) retrotransposons and non-LTR retrotransposons (Finnegan, 2012) depending on the transposition mode. The class II elements are called DNA elements containing terminal-inverted repeats (TIRs) and are subdivided into three groups in eukaryotes: classic transposons (Craig et al., 2015), helitrons (Kapitonov and Jurka, 2001), and mavericks, sometimes called politrons (Pritham et al., 2007). Transposons tend to modify the number of copies of genomic elements in the genome and, subsequently, could dysregulate gene expression, recombination, and chromosome crossing overs leading to chromosomal rearrangements. Thus, TEs are the major molecular mechanisms driving host genome evolution (Houe et al., 2019). The insertion of TEs into an exon may change the gene open reading frame (ORF) resulting in the coding of non-functional protein or in missense/non-sense mutations. However, transposition could also modify the alternative splicing and, therefore, the protein synthesis leading to produce protein isoforms or introduce polyadenylation signal, which both facilitate evolution and adaptation to environmental changes (Capy et al., 2000; Konkel and Batzer, 2010).

Comparison of *Ae. aegypti* and *Ae. albopictus* genomes, highlights a large difference in quantity and diversity of TEs elements; TEs cover 1,343 MB in *Ae. albopictus* and 988 MB in *Ae. aegypti*. In addition, 20% of TEs present in *Ae. albopictus* are absent in *Ae. Aegypti*, confirming the divergence of the two mosquito species 71 million years ago (Chen et al., 2015). Endogenous viral elements, TEs integrated into the DNA of germline, constitute the fossil records of past infections (Emerman and Malik, 2010). RNA viruses are characterized by a rapid rate of evolution close to 10^–3^ substitutions/site/year (s/s/y). However, once they are endogenized, the rate of evolution drastically declines to 10^–9^ (s/s/y) in mammals but remains relatively comparable in insects [10^–7^ (s/s/y)] (Duffy et al., 2008; Ballinger and Taylor, 2019).

Comparatively, *I. scapularis* genome is approximately 13 times bigger than *Aedes* mosquito genome (2.1 GB for *I. scapularis*) (Gulia-Nuss et al., 2016). This difference could be explained by the presence of repetitive DNA representing 70% of the genome and reflecting large accumulation of tandem repeats and TEs (Gulia-Nuss et al., 2016). However, high TEs quantity could be also explained by the time of divergence (∼ millions of years) between ticks and mosquitoes. Interfering RNAs actively modulates the activity of TEs, which may influence the competence of mosquitoes for arboviruses (Biryukova and Ye, 2015).

#### Close Genetic Interactions of Vectors and Viruses

Because most viruses transmitted by mosquitoes and ticks have positive sense RNA genomes, most integrated transposons in vector genome may belong to retrotransposons elements. However, the production of viral-derived double-stranded DNA (vDNA) is under the control of the RNA interference (RNAi) pathways, as suggested by the presence of vDNA in RNAi-deficient *Ae. albopictus* C6/36 cells compared to RNAi-competent *Ae. aegypti* Aag2 cells. Mosquito tolerance to high viral loads is believed to occur in *Aedes* mosquitoes by generating viral-derived DNAs, which impair vector immune responses (Goic et al., 2016). It was also reported that after infection of *Aedes* mosquitoes, vDNA had been found in wings and legs revealing the possible production and dissemination of vDNA from infected tissues or the production of vDNA by all mosquito cells (Goic et al., 2016). However, since non-retroviral viruses are not able to encode their own reverse transcriptase or integrase, they require endogenous enzymes to achieve transpositions. This critical process could be divided into three different steps: (i) reverse transcription of the non-retroviral RNA in vDNA, (ii) importation of these intermediates into the nucleus, (iii) and integration of non-integrated retroviral sequences (NIRVS) in the host genome (Hindmarsh and Leis, 1999). The initial step of transposition is the production vDNA from non-retroviral RNA virus, which surprisingly can only be partial, leading to the production of partial RNA genome. It is likely due to a switch of the reverse transcriptase from the original RNA template to a close viral genome leading to numerous reverse transcription events or to a misconduct of the reverse transcriptase (Geuking et al., 2009).

While integration of vDNA generated from DNA viruses have been largely described, little is known about integration of NIRVS into the host genome. Three different mechanisms allow the integration of vDNA from DNA viruses: non-homologous end joining (NHEJ) (Bill and Summers, 2004), non-homologous DNA recombination mediated by adeno-associated DNA virus (Deyle and Russell, 2009), or telomeric recombination (Morissette and Flamand, 2010). It has been reported that vDNA are produced early in mosquitoes following viral infection and are critical to trigger mosquito immune responses, leading to viral tolerance rather than viral resistance (Goic et al., 2016). More precisely, the production of vDNA, which has been detected in mosquitoes or in *Drosophila* after challenge with CHIKV or Flock House virus (FHV), respectively, promotes the viral tolerance (Goic et al., 2013). This process has been likely linked to RNAi pathways, considered as the most important immune pathways in arthropod vectors (Liu et al., 2019). One class of interfering RNA (PIWI)-interacting RNAs, are involved in regulating insertion of TEs (Arensburger et al., 2011; Akbari et al., 2013) and in mosquito antiviral defenses (see next section) (Morazzani et al., 2012; Vodovar et al., 2012). Interestingly, in mosquito and tick genomes, NIRVS are often located in clusters of this interfering RNA class (Olson and Bonizzoni, 2017; Palatini et al., 2017; Russo et al., 2019): 50% of NIRVS are integrated near this particular RNAi clusters in *Ae. aegypti*, 12.5% in *Ae. albopictus* (Ter Horst et al., 2019), and 99% in *I. scapularis* ticks (Russo et al., 2019), suggesting a potential link with P element-induced wimpy testis (PIWI)-interacting RNAs (piRNAs) pathway, making NIRVS a possible actor of antiviral response.

Finally, at the protein level, the NIRVS could be translated into proteins that may act as direct antiviral elements by affecting viral polymerase activity and blockade of viral replication (Fujino et al., 2014).

In addition to vector genome modifications, coevolution of vectors and the pathogens they transmit can positively modulate specific gene expression to maintain the vector fitness and secure pathogen transmission. In *Ae. aegypti* mosquitoes, a positive selection of RNAi genes [microRNA (miRNA) and small-interfering RNA (siRNA)] was observed in presence of DENV, since silent mutations of Dicer-1, Dicer-2, Ago-1, Ago-2, R3d1, and R2d2 genes were positively selected in field-collected mosquitoes (Bernhardt et al., 2012). It is now admitted that long-term contacts between vectors and pathogens presume strong molecular interactions, which allow efficient pathogen transmission with limited impact on vector fitness; this suggests a subtle balance between vector infection with a certain tolerance for the pathogen and the vector survival (Lambrechts and Saleh, 2019).

#### Molecular Interactions of Viruses With Vectors

##### Primary Defenses to Pathogen Infection

In mosquitoes and ticks, efficient transmission of pathogens corresponds to the successful crossing of different physical barriers that are midgut epithelium, hemocoel, and salivary glands, and the excretion of viral particles in saliva secreted during the feeding. First, the midgut includes different parts: the anterior region dedicated to the sugar absorption and the posterior part to the blood absorption. Upon acquisition of the blood meal, the midgut secretes a chitinous sac called the peritrophic matrix, which confines the blood meal facilitating the action of digestive enzymes. For example, in mosquitoes, some pathogens are able to modify the composition of the peritrophic matrix to more easily pass through it and infect the *Anopheles* mosquitoes (Dong et al., 2009). But independently of the vectors, infection of the midgut may depend on viral load of the blood meal absorbed by the vector (Kramer et al., 1981; Pesko et al., 2009). Second, after crossing the midgut epithelium, the pathogen disseminates into the hemocoel. There, immune cells named hemocytes are involved in pathogen recognition and elimination, similarly to macrophages in vertebrates by secreting pattern recognition receptors (PRRs), and to proteins involved in phagocytosis, nodulation, and melanization processes in arthropods. Hemocytes are also able to trigger signal transduction, stress response pathways, and produce antimicrobial peptides (AMPs) (Hernandez-Martinez et al., 2002; Castillo et al., 2006; Bartholomay et al., 2007). Third, the pathogen reaches the salivary glands where it replicates. By secreting the saliva during their blood feeding, vectors facilitate the uptake of blood and indirectly the transmission of pathogens. Vector saliva usually contains compounds to overcome host immune reactions by controlling local inflammation, cellular recruitment, and secretion of proinflammatory molecules by sentinel cells. Structurally, mosquito salivary glands are composed of lobes connected to a main salivary canal, whereas in ticks, they are grape-like and branched where different types of spherical acini (three types were described in Ixodid ticks, while in argasid tick, only two types are present) are directly attached either to a main or accessory salivary duct, which dump tick saliva into a single salivarium close to tick mouthparts. Some arboviruses including DENV-2 and CHIKV seem to exploit preferentially some lobes of mosquito salivary glands (Salazar et al., 2007; Tchankouo-Nguetcheu et al., 2012). To conclude, the efficiency of viral transmission clearly depends whether the viral load ingested during the blood meal is sufficient to overcome midgut barrier and primary vector’s responses to the viral infection first and then the capacity of novel virions to infect and replicate in salivary glands tissues (Paulson et al., 1989; Scott et al., 1990; Turell et al., 2006).

As the primary organ that intervenes in the blood digestion, the midgut plays a crucial role in the immune responses of vectors to pathogens. Various host-derived molecules named pattern recognition receptors (PRRs) bind to pathogens-associated molecular patterns (PAMPs). While most of PRRs are secreted proteins harboring adhesive domains interacting with the PAMPs, some others are intracellular such as Dicer-2 and cGAS (Martin et al., 2018), but all play a significant role in vector immune responses (Buchon et al., 2009; Sterba et al., 2011). Similar to vertebrates, arthropods have multiple protein families that can play PRRs’ role (Hajdusek et al., 2013; Kumar et al., 2018). Among them, the thioester-containing proteins (TEPs) are generally found in the hemolymph and are associated to pathogen neutralization in *Drosophila*, mosquitoes, and ticks (Lagueux et al., 2000; Cheng et al., 2011; Urbanova et al., 2015). Some proteins of this family act as phagocytosis enhancers as TEP1, also able to form with the LRIM and APL1C (leucin rich repeat proteins), a complex capable of binding bacteria and parasites in *Anopheles* mosquitoes (Fraiture et al., 2009; Povelones et al., 2009). However, ticks are unique invertebrates that harbor all the major classes of known TEPs both in vertebrate and arthropods including α-macroglobulins, C3-components of complement system, insect TEPs, and macroglobulin complement-related proteins (MCRs) (Buresova et al., 2006).

Another PRR-like molecule, the fibrinogen-related protein family (FREP), is particularly active in the maintenance of vector immune homeostasis and the degradation of various pathogens including bacteria, fungi, and *Plasmodium* (Ferguson and Read, 2002; Rego et al., 2006; Waterhouse et al., 2007). C-type lectins are also involved in the pathogen recognition both in the midgut and the hemocoel and are critical in antibacterial responses in mosquitoes (Osta et al., 2004; Schnitger et al., 2009). Finally, Gram-negative binding proteins (GNBPs), expressed in midgut, hemocytes, and salivary glands, are important in the immune responses to parasite and bacterial infections in mosquitoes (Dimopoulos et al., 1997; Warr et al., 2008).

**FIGURE 3:**
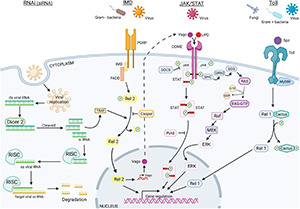
Vector immune pathways to fight against viral infections. The vector immune responses to pathogen infections, composed of four different pathways, allow vectors to neutralize entomopathogens, such as fungi, bacteria, or virus, and are involved in viral infection, replication, dissemination, and transmission along vector life cycle. siRNA (in green), JAK/STAT (in pink), IMD (in orange), and Toll (in blue) immune pathways are represented (Schonhofer et al., 2016; Terradas et al., 2017; Lee et al., 2019). Created with BioRender.com.

Finally, PRRs lead to the activation of all immune signaling pathways, to the production of AMPs such as defensins and lysozyme, and to the activation of the three main immune pathways: the Toll pathways, the IMD pathways, and the JAK/STAT pathway (Figure 3).

##### Mosquito and Tick Immune Pathways

*Toll Immune Pathway.* Both in ticks and in mosquitoes, this pathway is induced after fungi or Gram + bacterial infections, leading to the proteolytic cleavage of Spaëtzle ligand (Spz) that might also activate the nuclear factor kappa B (NF-kB) (Parker et al., 2001; Weber et al., 2003; Kumar et al., 2018). Then, the gene activation in the Toll pathway is controlled by a NF-kB transcription factor, Rel1. The Toll pathway activation leads to the production of AMPs including defensins, cecropins, gambicin, diptericin, and attacins (Chalk et al., 1995; Lowenberger et al., 1995; Cho et al., 1996; Xiao et al., 2014). The melanization process is dependent on serine protease, serpins, and phenoloxydase and corresponds to an enzymatic cascade, which ends up killing the pathogen by surrounding it with a layer of chitin-restricting nutritive uptake; the signal is given by the increase in reactive oxygen species (ROS). In mosquitoes, the Toll pathway can also be repressed by a negative regulator, Cactus, while ticks have two supplementary negative regulators named TOLLIP and SARM (Fogaca et al., 2021). Besides, it is clearly established that, in mosquitoes, the Toll pathway is important in the regulation of viral infection, as observed for DENV in *Aedes* (Xi et al., 2008) or for ONNV in *Anopheles* (Waldock et al., 2012). In ticks, this pathway is not fully characterized, but several studies corroborate the importance of Toll pathway in the regulation of viral infection as flavivirus infections upregulated Toll genes (Mansfield et al., 2017).

*Immune Deficiency Pathway*. The immune deficiency (IMD) pathway overlaps the responses triggered by the Toll pathway, such as the melanization and the production of AMPs including cecropin1. The activation of this pathway also requires the binding of PRRs by Gram-negative bacteria (Leulier et al., 2003) or viruses (Costa et al., 2009). Similarly to the Toll pathway, Rel2 is a protein belonging to the family of NF-kB transcription factors; it activates the IMD pathway modulated by the negative regulator Caspar. However, IMD pathway activation is also regulated directly by midgut microbiota, which plays a critical role both in the vector and pathogen transmission (Ramirez et al., 2012).

In mosquitoes, it was observed that a blood meal leads to the activation of the IMD pathway through nutriment and induces growth of microbiota, which ends to the upregulation to the Rel2 protein, likely to control microbiota levels in the midgut facilitating viral infection (Barletta et al., 2017). This upregulation of Rel2 and the IMD pathway activation negatively modulates the malaria parasites in anopheles mosquitoes (Meister et al., 2009).

Interestingly, genomic studies revealed that ticks lack most of the *Drosophila* orthologs described as acting in IMD pathway such as the peptidoglycan recognition proteins (PGRPs), the Fas-associated with death domain (FADD), the adaptor molecule IMD, and the death-related ced-3/Nedd2-like protein (DREDD) (Fogaca et al., 2021). The absence of these defense orthologs is apparently not restricted to ticks, since it is also reported for arachnids or hemipterians (Palmer and Jiggins, 2015; Nishide et al., 2019) but may be mainly due to the limited genomic data available (Gulia-Nuss et al., 2016). Nevertheless, in ticks, as in other vectors, the IMD pathway is strongly activated after bacterial infection and recognition by the PGRPs of *A. phagocytophilum* and *B. burgdorferi* (Shaw et al., 2017). After bacteria recognition, the X-linked inhibitor of apoptosis (XIAP) complexes with Bendless and ubiquitylates its P_47_ substrates leading the phosphorylation of the IKKβ, an inhibitor of NF-kB kinase. Then, Rel2 is translocated into the nucleus. Likewise, growth factor-β-activated kinase 1 (TAK1) and TAK1 adaptor protein 1 (TAB1) may also activate the ortholog JNK signaling pathways as in *Drosophila* (Silverman et al., 2003).

*JAK/STAT Pathway*. The JAK/STAT pathway is composed of an unpaired peptide ligand (Upd), a transmembrane protein receptor (Dome), Janus kinase (JAK), and STAT proteins. The binding of Upd to the extracellular terminal of the Dome induces JAK/STAT pathway receptors, then initiating the dimerization of these receptors and the phosphorylation of JAK associated with receptor dimers. Then, activated JAK phosphorylates the receptor dimers C-terminus, inducing the production of binding pockets where STAT proteins are phosphorylated by the JAK–Dome complex, resulting in both activation and dimerization of the STAT. Once activated, STATs are translocated into the nucleus; target genes are transcriptionally regulated (Agaisse and Perrimon, 2004). The JAK/STAT implication was already confirmed in the DENV replication in *Ae. aegypti*, as the silencing of the inhibitor of activated STAT decreased viral replication, and conversely, the silencing of the receptor JAK increased viral replication (Jupatanakul et al., 2017).

In ticks, the 5,3-kDa AMP is regulated by the JAK/STAT pathway and plays an important role in limiting *A. phagocytophilum* infection in tick salivary glands (Liu et al., 2012) and *B. burgdorferi* infection (Ribeiro et al., 2006), or LGTV infection (McNally et al., 2012). *I. scapularis* hijacks immune molecules secreted by vertebrates by stimulating the JAK/STAT pathway, the interferon gamma (IFN-γ) acting as an effector of the tick pathway and the production of AMPs (Smith et al., 2016; Capelli-Peixoto et al., 2017).

More recently, the JAK/STAT pathway has been found to be activated not only by pathogens but also *via* oral absorption of nutrients, and especially blood compounds. Initially identified in *Drosophila*, the extracellular signal-regulated kinase (ERK) pathway was documented as mechanistically linked to nutrient uptake and antiviral innate immunity in insects (Christophides et al., 2002; Katsuma et al., 2007; Sabin et al., 2010). In blood ingested by hematophagous insects, growth factors such as insulin are shown to trigger ERK signaling in the mosquito gut (Surachetpong et al., 2009). The insulin peptide was identified in different mosquito tissues (including head, thorax, and abdomen) in its integral form for at least 48 h after blood ingestion (Drexler et al., 2013). Triggering ERK pathway restricts several viral infections including human arboviruses; the canonical ERK signaling components dSos, dRas (Ras85D), dMek (Dsor1), dErk (rl), ksr, and cnk regulate insulin sensitivity (Zhang et al., 2011) and arbovirus infection (Xu et al., 2013). Activation of the ERK pathway resulting in an increase in phosphorylated ERK can take place < 30 min after the exposure to the insulin stimulus (Kang et al., 2008; Surachetpong et al., 2009). The ERK signaling pathway restricts orally acquired viral particles in enterocytes of the mosquito digestive tract. The antiviral action seems to be triggered independently of the RNAi pathway (Ahlers et al., 2019). Moreover, the microbiota may affect the insulin response of vectors; *Wolbachia* downregulates *Aedes* InR expression and reduces DENV and ZIKV replication in mosquitoes (Haqshenas et al., 2019).

*RNA Interference*. Compared to vertebrates, invertebrates lack adaptive immunity, which produce effective response to viral infection. RNAi pathway could, from a certain point of view, be considered as adaptive immune response, since the siRNAs produced target specifically nucleic acids of the pathogen genome. First described in plants, siRNAs are the hallmark of antiviral RNAi response (van Rij and Andino, 2006; Ding and Voinnet, 2007; van Rij and Berezikov, 2009). The RNAi pathway was later deciphered in *Drosophila melanogaster* (Galiana-Arnoux et al., 2006; Wang et al., 2006), and the RNAi pathway inhibitor FHV B2 was discovered in flies inoculated with *Drosophila* C virus (DCV) (Cherry and Perrimon, 2004). The RNAi pathway is physiologically activated by double-stranded RNA (dsRNA), leading to the production of small RNAs harboring different features. First, the endogenous siRNAs initially discovered in *C. elegans* in 1995 (Guo and Kemphues, 1995), are naturally involved in cellular process regulation in addition to play critical roles in antiviral immunity by processing the exogenous viral RNA. This pathway is present not only in mosquitoes but also in other arthropods as ticks where orthologs play the same role that of Dicer2, R2D2, and Ago2 (Argonaute-2) (de la Fuente et al., 2007; Belles, 2010). Briefly, the dsRNA viral replication intermediates are recognized by Dicer-2 *via* the RNA-binding site and then cleaved into siRNAs of 22nt length fragments. The antisense strand binds to the RNA-induced silencing complex (RISC) composed by TRBP, Ago2, and Dicer, which select the guide strand. The activation of the RISC complex by the C3PO enzyme in the cytoplasm ends with the degradation of the complementary viral RNA sequence to RNA guide. Second, miRNAs are involved in the regulation of endogenous gene expression. While the siRNA pathway occurs in the cytoplasm, the miRNA pathway has both nuclear and cytoplasmic phases (Donald et al., 2012). Their structural stem loop allows their processing by Drosha (in the nucleus), which results in the production of miRNA precursors (Yeom et al., 2006). Once in the cytoplasm, these precursors are processed by Dicer-1 cutting their loop, creating miRNA duplex, which is charged on the RISC complex. From there, processes of miRNA and siRNA are similar with the formation of the RISC complex, the production of the RNA guide serving to target the mRNA and to affect the gene expression. Many replication and dissemination of arboviruses in mosquitoes are controlled by these pathways, as it was reported for ONNV in *Anopheles* (Keene et al., 2004), CHIKV in *Aedes* (McFarlane et al., 2014), or flaviviruses such as ZIKV and DENV, and in tick-borne flavivirus Langat virus (Sanchez-Vargas et al., 2009; Schnettler et al., 2014; Saldana et al., 2017). Nevertheless, the role of miRNA in the antiviral responses in ticks remains elusive. The piRNAs, mainly known for its role in the germ line protection from TEs insertion, are present in mosquitoes and ticks (Arensburger et al., 2011; Akbari et al., 2013). However, this RNAi pathway is Dicer independent and causes gene silencing by antisense binding. The antisense transcript arose from piRNA clusters and is loaded on the PIWI protein, which, in the cytoplasm, interacts with the endonuclease to process the 3′ end of the piRNA. They are cleaved, loaded on the complex, and transported back to the nucleus (Ross et al., 2014). An alternate mechanism leads to selective amplification of piRNA, called the ping-pong cycle, and targets TEs. However, the piRNA from the primary pathway combines with the PIWI protein forming the PIWI/piRNA complex, which binds to the TEs and leads to the cleavage of piRNA (Kumar et al., 2018). In ticks, as in non-mosquito arthropods, piRNAs are very likely present, but it is still unclear whether they contribute to the antiviral mechanism (Russo et al., 2019; Talactac et al., 2021).

Finally, pathogen–vector interactions are really complex and involve past genetic elements as NIRVS and TEs, which occurred during the natural history of the vector and are inserted in the vector genome. Past common history of vectors and pathogens influences the outcome of vector infection by a pathogen. Thus, vector genetics appear to be one of the main determinants controlling their interactions. Vector genetic factors involved in the viral transmission resulting in long-term interactions appeared as critical for the success of pathogen transmission, but closer environment is also determinant as we will demonstrate just after.

### Non-genetic Factors Modulating Viral Transmission

Throughout their life, arthropod vectors directly interact with their environment since their feeding and reproduction require, respectively, the presence of hosts and specific locations to lay eggs. In addition, symbiotic microorganisms, mainly commensals, colonize the vector during immature lifecycle stages modifying vector metabolism, behavior, and immune system. It has been proven that these interactions can consequently impact pathogen transmissions.

#### Role of Microbiota

The microbiota represents a set of microorganisms living in symbiosis within the vector, which can affect pathways such as blood digestion, reproduction, general metabolism, and innate immunity of vectors (Engel and Moran, 2013). In arthropods, microbiota evolves throughout the life of the vector (Colman et al., 2012; Zolnik et al., 2018). Mosquito gut microbiota varies a lot not only between the aquatic and terrestrial phases but also between males and females. In *An. gambiae*, the bacterium *Cyanobacteria* predominates in the midgut of immature stages (40%), while at the adult stage, *Proteobacteria* and *Bacteroidetes* are the most abundant (up to 61.7%) (Wang et al., 2011). Female mosquitoes are hematophagous, while males mostly feed on flower nectar. This diet difference impacts the composition of the gut microbiota because, in females, the oxidative stress produced by blood assimilation releases ROS that is generally toxic and results in an indirect selection of the gut flora. Thus, the bacterial diversity of female mosquitoes is reduced compared to males and is mainly composed of *Enterobacteriaceae* (resistant to this environment) (Wang et al., 2011). In *Ae. albopictus* females, a majority of *Proteobacteria* is observed, whereas in males, *Actinobacteria* is dominant, which could be explained by the different sources of flower nectar absorbed by males (Valiente Moro et al., 2013). In ticks, characterization of the role of the microbiome in pathogen transmission is less studied than in mosquitoes, although its composition is becoming better determined (Narasimhan and Fikrig, 2015; Aivelo et al., 2019). For example, in the tick midgut, the main genera identified were *Acinetobacter* spp., *Enterorobacter* spp., *Sphingobacterium* spp., *Pseudomonas* spp., and *Stenotropomonas* spp. (Narasimhan and Fikrig, 2015). As demonstrated, the microbiota composition depends on which organs of the tick are studied, since the well-known *Wolbachia* spp. have been isolated from ovaries and salivary glands as for mosquitoes, but not in the gut. Moreover, it has been shown that many parameters modulate the tick gut microbiota such as geographical location, developmental stages, or even the feeding mode (Van Treuren et al., 2015; Berhanu et al., 2019).

The vector microbiome affects the transmission of certain pathogens such as bacteria, parasites, or arboviruses. Therefore, midgut microbiota takes part in vector infection and subsequently in dissemination and transmission. As previously mentioned, the typical bacteria-colonizing vector guts are *Wolbachia* spp., which are found in over 66% of arthropods (Hilgenboecker et al., 2008). It is detected in many vectors of arboviruses such as *Ae. albopictus*, *Ixodes* spp., or *C. quinquefasciatus* (Hilgenboecker et al., 2008). Interestingly, two phylogenetically close mosquito species, *Ae. albopictus* and *Ae. aegypti* present distinct profiles of gut microbiota: *Wolbachia* is present in *Ae. Albopictus*, while it is absent in *Ae. aegypti*. *Wolbachia* is an intracellular bacterium located in the cytoplasm of many cells such as intestinal or ovarian cells. It has been shown that the presence of this bacterium may limit the transmission of arboviruses such as DENV, ZIKV, and YFV and also *Plasmodium* parasites (Brownstein et al., 2003; Moreira et al., 2009; van den Hurk et al., 2012). Interestingly, the level of antiviral inhibition depends on the density of *Wolbachia* in mosquito tissues as observed in DENV-infected *Ae. albopictus* Aa23 cells with high bacterial densities where the DENV was reduced compared to *Wolbachia*-cured cells (Rainey et al., 2014). Due to its importance in pathogen transmission, *Wolbachia* is one of the most promising ways to replace insecticides in vector control (McGraw and O’Neill, 2013).

In *Ae. albopictus*, *Wolbachia* induces distortions of host reproduction *via* a form of sterility known as unidirectional cytoplasmic incompatibility, which make sterile any *Wolbachia*-free females mating with a *Wolbachia*-infected male (Blagrove et al., 2012). Thus, the transmission of arboviruses is altered by the decrease in mosquito population.

Vector microbiota (including *Wolbachia*) triggers basal expression of the Toll pathway, consequently reducing the vector capacity to transmit arboviruses as observed for DENV in *Ae. aegypti* (Rances et al., 2012). Besides its role in immune responses in order to maintain host homeostasis, autophagy may also be corrupted by some viruses to complete some proviral roles (Sinkins, 2013). As an example, DENV requires specifically autophagy linked to lipid droplets for optimal viral replication in mammalian cells. DENV-induced autophagolysosomes are found to colocalize with lipid droplets to become autolysosomes, generating free fatty acids (Samsa et al., 2009). However, lipid droplets are suspected as involved in DENV particles assembly, which requires free fatty acids (Perera et al., 2012). However, when Wolbachia-B is inoculated into *Ae. aegypti*, DENV replication is strongly inhibited, suggesting a possible competition for host resources between virus and bacteria. It seems that *Wolbachia* competes for cellular resources with viruses to grow/replicate through autophagy manipulation/modulation for cholesterol supply (Moreira et al., 2009).

In the midgut, the bacterial flora competes with the pathogens ingested during the blood meal for nutritional resources (especially lipids, including cholesterol). *Wolbachia* and *Spiroplasma* and also other bacteria could modify lipid metabolism or sequester cholesterol, which is critical for the formation of enveloped virion, thus restricting viral replication (Yin et al., 2020). Thus, the presence of bacteria in the midgut challenges the vector innate immunity or even modulates vector metabolism, which may influence the transmission of arboviruses (Jupatanakul et al., 2014; Hegde et al., 2015; Bonnet et al., 2017; Gao et al., 2020).

Finally, microbiota may have direct effects on arbovirus by secretion of secondary metabolites, which are molecules produced by bacteria involved in survival, fecundity, or defense, providing a selective advantage. The implication of these metabolites was suggested since half of bacteria from *Ae. albopictus* gut caused a reduction up to 44% of La Crosse Virus (LACV) infectivity on Vero cells. Among these bacteria, *Pseudomonas rhodesiae*, two *Enterobacter ludwigii*, and *Vagococcus salmoninarium* exhibited the highest reduction effect (Joyce et al., 2011). Similarly, *Chromobacterium* (Csp_P) isolated from the midgut of field-caught *Ae. aegypti* and able to make biofilms totally inhibits DENV in BHK cells, suggesting that the biofilm formed by Csp P after 48 h of growth produced molecules with antiviral properties (Jupatanakul et al., 2014).

Just as some bacteria are able to create symbiotic interactions with vectors, several viruses are similarly able to persist sustainably in mosquitoes and ticks. These arthropod-specific viruses (ASV) replicate only in invertebrate cells, not in vertebrate cells as arboviruses do. These specific viruses may modulate virus transmission by the vectors.

#### Coinfections of Arthropod-Specific Viruses With Arbovirus

The ASVs are a particular class of viruses that are only able to replicate in arthropod cells, not in other cell lines. Initially discovered 45 years ago in *Ae. aegypti* cells, the first ASV was an insect-specific virus (ISV) named cell fusing agent virus (CFAV) in reference to the syncytia formed in infected cells, which belongs to the *Flaviviridae* family and was identified in many mosquito populations around the world. This finding suggests that it represents a possible ancestral lineage of flaviviruses (Stollar and Thomas, 1975; Marin et al., 1995). The Kamiti River virus (KRV) also belonging to the *Flaviviridae* family was discovered many years later from *Ae. macintoshi* mosquitoes collected in Kenya. While it is genetically and phenotypically close to CFAV, it differs by the absence of syncytia formation in infected cells (Crabtree et al., 2003).

Over the past 10 years, the ISVs have been extensively studied, identified, and characterized. To date, more than 60 ISVs have been identified and belong to the following viral families: *Flaviviridae*, *Togaviridae*, *Rhabdoviridae*, *Bunyaviridae*, *Reoviridae*, *Mesoniviridae*, *Tymoviridae*, *Birnaviridae*, *Negeviruses*, and *Nodaviridae* (Bolling et al., 2015). Among them, some of these ISVs have been demonstrated to strongly reduce arbovirus transmission (Bolling et al., 2015). The flaviviruses Nhumirim virus (NHUV) isolated from *Culex chidesteri* and Palm Creek virus (PCV) isolated from *Coquillettidia xanthogaster* can reduce or completely abolish the replication of few flaviviruses such as JEV, WNV, and Saint Louis encephalitis virus (SLEV) for the first one and Murray Valley Encephalitis Virus (MVEV) and WNV for the second one on C6/36 cells (Bolling et al., 2012; Kenney et al., 2014; Pauvolid-Correa et al., 2015). In addition, the presence of NHUV also diminished the flaviviruses JEV and SLEV load in arthropod cells (Kenney et al., 2014). Similarly, the Culex flavivirus, CxFV, decreased WNV dissemination from 94 to 72% in *Culex pipiens* colony at 7 days postinfection (Hoshino et al., 2007; Bolling et al., 2012). Alone, the ISVs are capable of reducing or blocking viral transmission; thus, when mosquitoes are infected with multiple ISVs including CxFV, PCV, or NHUV, a reduction in WNV transmission was observed (Bolling et al., 2012; Hobson-Peters et al., 2013; Kenney et al., 2014). Likewise, the coinfection of CFAV and Phasi Charoen-like virus (PCLV) interferes with the replication of ZIKV and DENV and inhibits the infection of La Crosse virus (LACV) in *Ae. albopictus* cells (Schultz et al., 2018). Similarly, the LACV replication was also greatly diminished upon *Aedes* cells coinfected with CFAV and PCLV (Schultz et al., 2018). However, many of these studies were performed in *Ae. albopictus* C6/36 cells that are RNAi deficient.

Mechanisms underlying the disruptions of arboviral transmission by ISV are still poorly understood. Some hypotheses suggest that interference occurs when only ISV and arbovirus belong to the same viral family, as it has been observed with LACV and PCLV, two bunyaviruses having similar viral cycles (Schultz et al., 2018). However, some exceptions exist as has been shown in *C. quinquefasciatus*; when infected with CxFV (*Flaviviridae*), the replication of WNV (*Flaviviridae*) was inhibited (Kent et al., 2010; Crockett et al., 2012). Conversely, an infection with DENV and JEV was not reduced in a *Culex tritaeniorhynchus* cell line (CTR cells) infected with CxFV (Kuwata et al., 2015). These data clearly suggest that the ISV–arbovirus interactions seem to be mostly specific to the virus and mosquito species.

Conversely to mosquitoes, the identification of ASV in ticks is less advanced due to the limited knowledge on these vectors mainly focused on the tick-borne viruses (TBVs) rather than tick-specific viruses (Calisher and Higgs, 2018). However, more and more viruses composing the tick virome are discovered (Pettersson et al., 2017; Vandegrift and Kapoor, 2019). Some invertebrate viruses are identified in *I. scapularis* ticks ISAV-1 and ISAV-2 (*I. scapularis*-associated virus 1 and 2) with the highest similarities to *Sobemovirus* genus, a single-stranded positive-sense RNA virus that infects plants. Similarly, in *I. ricinus* collected in Norway, some viruses belonging to *Bunyaviridae*, *Luteoviridae*, *Mononegavirales*, and *Partitiviridae* families were identified as close to previously characterized viruses in *I. scapularis* (Tokarz et al., 2014). In *I. scapularis* IDE2 cells, a tick-specific orbivirus (*Reoviridae* family), the Saint Croix River Virus (SCRV), has been detected (Attoui et al., 2001; Nuttall, 2009; Bell-Sakyi and Attoui, 2013). In addition, in *D. variabilis* ticks, a *Omegatetravirus* genus-like (*Alphatetraviridae* family) was identified in only 11% of tested ticks with very low amino acid similarities (<19%). This virus is usually isolated from moths (order Lepidoptera) (Tokarz et al., 2014). As for mosquitoes, the possible link between tick-specific viruses and tick-borne virus transmission, is increasingly understood (Vandegrift and Kapoor, 2019).

Coinfections of arboviruses and ASV are henceforth established as limiting factors for mosquito and likely tick infections. However, these arthropod-specific viruses are not the only ones that may limit arthropod infection. Coinfections with different arboviruses can also occur, either simultaneously or sequentially in mosquitoes. This may result in various types of interactions: competition, cooperation, or neutral coexistence.

#### Coinfections of Arboviruses

Owing to globalization, co-circulation of arboviruses in the same region is quite common as observed for JEV and DENV in Asia, DENV and YFV in Africa, and ZIKV, DENV, and YFV in South America (Oladipo et al., 2018; Kayiwa et al., 2019; Saxena et al., 2019; Vogels et al., 2019). Therefore, a vertebrate host can be coinfected by two or more arboviruses. For example, in Brazil, 12 people were described as coinfected with both DENV and ZIKV during the dengue outbreak in 2016 (Estofolete et al., 2019). A mosquito can be infected with multiple arboviruses (acquired simultaneously or sequentially) and presumably transmit the different viruses in a single bite (Vogels et al., 2019). It was demonstrated that both *Ae. aegypti* and *Ae. albopictus* were able to cotransmit DENV and CHIKV after sequential infections with both viruses (Nuckols et al., 2015). Similarly, *Ae. aegypti* mosquitoes coinfected with any of CHIKV, ZIKV, and DENV-2 combinations could transmit all viruses whatever the combination (Ruckert et al., 2017). However, if such coinfections can occur in vectors, it is likely that, in some cases, the presence of an arbovirus in the mosquito limits the transmission of a second arbovirus, as it was observed with ISVs. Indeed, in C6/36 cells infected with DENV and then superinfected 7 days later with YFV, a significant decrease in YFV replication was detected (Abrao and da Fonseca, 2016). Similarly, a successive infection with YFV and DENV-2 at day 7 also led to a decrease in DENV-2 replication, suggestive of a blocking mechanism that is provoked by the first flavivirus infection (Abrao and da Fonseca, 2016). Mechanisms underlying the blocking of one arbovirus by another are not well understood yet and deserve to be further investigated. It has been suggested that an arbovirus primoinfection could trigger the host immune system, described as immune priming, hindering the replication of a second ingested arbovirus (Abrao and da Fonseca, 2016).

As demonstrated until now, the internal factors including vector genetics and epigenetics play critical roles in the vector infection, dissemination, and transmission of pathogens. However, factors related to the nature of the vector, their habitat (which impacts the vector microbiome), the class of pathogen they can host, and the history of past viral infections could also modulate vector infection. Furthermore, internal factors alone are not sufficient to explain the specific interactions between the vector and the pathogen, suggesting that other factors may intervene such as the direct and global environment.

## Role of External Factors in Virus Transmission by Mosquitoes and Ticks

Modulation of the vector capacity is, as described above, driven by vector internal parameters that compose the vector competence and by vector–environmental interactions. These interactions between the vector and living organisms in the vector ecosystem (plants, vertebrates, etc.) are named biotic factors. Conversely, the abiotic factors represent the relations of vectors with their physicochemical environment.

### Biotic Factors

Biotic factors refer to effects of living organisms interacting within an ecosystem. Among them, predation, intra-, and interspecific competition, parasitism (entomopathogenic parasites), and availability and quality of food resources are some examples. Here, we will focus on how vector diet, food habit, and breeding site composition can influence pathogen transmission.

Diet is an important factor that can affect many mosquito traits such as longevity, frequency of bites, reproduction, and susceptibility to pathogens (Carvajal-Lago et al., 2021). Recently, it was shown that *Anopheles coluzzii* fed on papaya nectar lived longer and had better mating rates than those fed on banana nectar (Nignan et al., 2020). In addition, *Culex pipiens* fed with low sugar content solutions (2 and 10% sucrose) were more likely to transmit WNV than those fed on high sugar diet (40%). Furthermore, the nutritional deficiency caused by low sugar diet decreased mosquito energy and fitness and provoked nutritional stress, thus favoring the viral infection (Vaidyanathan et al., 2008). In addition, the blood source may influence pathogen infection and transmission by vectors mainly by modulating cellular responses and immune priming. First, the degradation of ingested hemoglobin (Hb) can yield the secretion of antimicrobial peptides in mosquitoes and ticks (Sojka et al., 2013; Pakpour et al., 2014). Hb degradation also catalyzes the synthesis of ROS, which may favor parasites development in mosquitoes (Peterson et al., 2007). Insulin/insulin-like growth factor highly conserved in arthropods (Pakpour et al., 2014) is involved in different immune pathways (Luckhart and Riehle, 2007); insulin inhibits RNAi pathway but activates JAK/STAT pathway eliciting antiviral effects in mosquitoes following infection with WNV, DENV, and ZIKV (Ahlers et al., 2019). Transforming growth factor beta 1 (TGF-β1) from mammalian blood also regulates the production of the antiviral nitric oxide (Kreil and Eibl, 1996; Lin et al., 1997). Moreover, IFN-γ is able to activate IFN-dependent pathway in arthropods including the JAK/STAT pathway.

Pathogen transmission is also influenced by vector biology, especially at the interface of vector and the vertebrate host. As pool feeders, ticks, and particularly hard ticks, may have very long blood feeding depending on life stages (from days to weeks), allowing important blood absorption (up to 100 times their weight). Since they alternatively ingest blood and secrete saliva, they inject pathogens all along their long blood meal facilitating pathogen transmission. Indeed, as main tick genera of public health importance, *Ixodes*, *Dermacentor*, *Amblyomma*, or *Rhipicephalus* (excepted *R. annulatus*) has a three-host life cycle, meaning that each development stage (larva, nymph, and female) feeds on different vertebrate hosts (Centers for Disease Control and Prevention, 2017). *Hyalomma* ticks alternate between two hosts during their life cycle, while *Ornithodoros* are multihost argasid ticks. This host alternation also promotes pathogen transmission from one vertebrate to another. Tick cofeeding is characterized by pathogen transmission between infected and non-infected vectors that feed in close spatiotemporal proximity on the same host that has not yet developed a systemic infection. At the bite site, some tick-borne pathogens, including viruses, are thus able to rapidly pass from infected ticks to pathogen-free ticks through blood and lymph (Gordon et al., 1993; Labuda et al., 1993; Randolph et al., 1996). This phenomenon is not documented for mosquitoes, as they feed directly from capillaries and not from a hemorrhagic pool.

The last factor impacting the pathogen transmission is the breeding site, especially for mosquitoes, as their life cycle consists of both aquatic and terrestrial phases. The nature and composition of mosquito breeding sites influence the growth, lifespan, microbiota, and transmission of pathogens at the adult stage (Dickson et al., 2017; Hery et al., 2021a, b). In fact, a nutrient-poor breeding site may weaken the mosquito immune system, which may promote transmission of arboviruses in adults. Indeed, food starvation of *Ae. aegypti* larvae was demonstrated to increase infection rate (from 37 to 55%) and dissemination rate (from 26 to 45%) of Sindbis virus in adult mosquitoes (Muturi et al., 2011).

### Abiotic Factors

In their natural habitats, vectors are constantly exposed to different environmental factors affecting the vectorial system, creating favorable or unfavorable conditions to vector transmission. Abiotic environmental factors assemble all physicochemical parameters of an ecosystem and include climatic, chemical, and topographical factors, the latter affecting significantly VBDs.

#### Impact of Climatic Factors on Vectorial Transmission

Due to the seasonality of many VBDs, a close relation between the occurrence of VBDs and climate has been underlined (Lord, 2004; Altizer et al., 2006; Grassly and Fraser, 2006). Rainfall, humidity, photoperiod, and temperature are important climatic variables that affect either directly or indirectly not only various aspects of vector biology (development, survival, longevity, distribution, and seasonality) but also replication and transmissibility of viruses (Costanzo et al., 2015; Burtis et al., 2016; Young, 2018; Bellone and Failloux, 2020). Rainfall is involved in formation and persistence of mosquito breeding sites and thus conditions mosquito densities (Ho et al., 1971; Fouque and Reeder, 2019). Different studies showed a positive correlation between rainfall and the incidence of chikungunya in India (Shil et al., 2018) and that of dengue in the Philippines (Su, 2008). Rainfall also affects tick population, since these vectors, hard or soft tick species, are very dependent on local hygrometry to survive in their respective habitats. In Australia, Argentina, and Kenya, global modifications of tick habitats through the modification of seasonality and intensities of rainfalls and temperature raising increase the densities of the cattle tick *Boophilus microplus*, and losses in beef cattle industry are expected (White et al., 2003; Estrada-Pena et al., 2006; Keesing et al., 2017). Humidity is well known for promoting mosquito and tick survival, therefore enhancing their chance to transmit pathogens (White et al., 2003; Schmidt et al., 2018). In Vietnam, dengue epidemics have been demonstrated to be closely linked to increases in rainfall and humidity; the incidence of dengue fever increased by 1% for every 50 mm increase in rain water and 1% of humidity (Xuan le et al., 2014). In addition, low local humidity indirectly influences TBP, as the climate and environment directly affect tick-questing behavior and abundance. However, in temperate regions, tick abundance is probably more related to the host availability than to climate variations (Paul et al., 2016). Photoperiod is another parameter that profoundly affects vectors’ life traits (Costanzo et al., 2015). For the mosquito *Ae. albopictus*, a drop of photoperiod in autumn induces the production of diapausing eggs, signing the end of mosquito adult activities and the period suitable for pathogen transmission (Armbruster, 2016). However, in tropical regions, the mosquito *Ae. aegypti* can also respond to photoperiod changes; females subjected to photoperiod reductions survived longer and blood fed more frequently than females exposed to longer photoperiods (Costanzo et al., 2015). Interestingly, ticks are also very dependent on the photoperiod, which rhythms their activity and rest periods in nature. Prolonged photoperiod affects soft tick mortality, with up to 36% of mortality in *Ornithodoros turicata.* Surprisingly, the opposite effect was observed on the progeny of *O. turicata* that was continuously reared in the dark (Adéyeyè and Phillips, 1996); larvae in continuous darkness gained more weight than those reared under standard conditions (Adéyeyè and Phillips, 1996). The long photoperiod also shortens the tick life cycle by reducing molting time. But conversely, ticks exposed to long photoperiod begin their oviposition later than those exposed to short photoperiod or continuous darkness (Adéyeyè and Phillips, 1996).

Lastly, temperature is one of the most important abiotic factors, affecting significantly vectors and the pathogens they transmit (Samuel et al., 2016). Because arthropods are poikilothermic ectotherms, many of their life traits including egg viability, development of immature stages, adult survival, behavior, and physiology (i.e., microbiota and immune responses) strongly depend on environmental temperature (Murdock et al., 2012; Lefevre et al., 2013; Narasimhan and Fikrig, 2015; Thapa et al., 2019; Bellone and Failloux, 2020). Along with the photoperiod, seasonal temperature oscillation is a critical factor for tick activity and, subsequently, pathogen transmission. Temperature increases after the winter period associated with longer photoperiod, speeds up egg hatching, oviposition, and molting. The questing behavior of nymphs and females is also positively affected by warmer temperatures (Randolph et al., 2002; Li et al., 2016).

Likewise, the replication and transmission of many pathogens including arboviruses are widely temperature dependent (Bellone and Failloux, 2020). Higher temperatures can shorten the vector developmental cycle (Delatte et al., 2009) and the extrinsic incubation period, the time required for vectors to become infectious following the ingestion of an infected blood meal (Liu et al., 2017; Wimalasiri-Yapa et al., 2019; Winokur et al., 2020), all increasing vectorial capacity. However, in a more subtle way, higher temperatures can also reduce vector lifespan (Estrada-Pena et al., 2012; Brady et al., 2013), which decreases the vectorial capacity.

The impact of climate change, especially global warming, on VBDs has become the topic of intense debate (Hongoh et al., 2012; Morin and Comrie, 2013; Caminade et al., 2019). Climate change has already favored the mosquito species *Ae. albopictus* to settle in temperate regions (Caminade et al., 2012). Less than 30 years after its first detection in Europe, *Ae. albopictus* has been incriminated in local transmission of DENV, CHIKV, and ZIKV (Bellone and Failloux). Another species, *Cx. tarsalis*, has spread over an area that is 1.06–2.56 times its current distribution and 1.08–2.34 times, the current geographic area of WNV it transmits (Chen et al., 2013). Likewise, different tick species are likely to establish more northern permanent populations in a climate-warming scenario (Gray et al., 2009); *Ixodes ricinus* expansion has been accompanied by an increased prevalence of tick-borne encephalitis. Collectively, it appears obvious that climate change, if not mitigated or properly managed, is very likely to broaden the geographic range of some VBPs, thus exposing human populations to higher risk for VBDs (Githeko et al., 2000; McMichael et al., 2006; Gould and Higgs, 2009; Semenza and Suk, 2018). However, caution should be taken with uncertainties of some prediction models neglecting the complex interactions between pathogens, vertebrate hosts, vectors, and the environment (Sutherst, 2004; Reiter, 2008; Tabachnick, 2016).

#### Chemical Factors

The chemical composition of breeding sites conditions the choice of mosquito laying site, impacting larval development and mosquito survival (Hershey et al., 2010; Matthews et al., 2019; Hery et al., 2021a, b). In urban parks in São Paulo, Brazil, type and pH of larval habitats were the best predictors of *Ae. albopictus* presence and abundance (Medeiros-Sousa et al., 2020). For *Ae. aegypti*, pH and salinity were the best predictors of mosquito abundance, while dissolved oxygen and type of larval habitat were better predictors of presence of mosquito species (Medeiros-Sousa et al., 2020). Other factors such as concentration of mineral elements, especially heavy metals like iron, zinc, and copper, are also important, especially in areas disrupted by human activities (Jeanrenaud et al., 2020). As the tick’s life cycle does not pass through an aquatic phase, it is likely that ticks are less sensitive to chemical compounds in their environment than mosquitoes.

#### Topographic Factors

Altitude is a topographic factor that can be used as a proxy of vector transmission risk. Increase in altitude is associated with different ecological factors critical for vector development, in particular temperature. Above 1,600 m, *Ae. aegypti* occurrence is predicted in < 1% of the total land area of 16 countries in America. Across all 16 countries, only 1.1% of historical dengue cases were reported above 2,000 m, suggesting that the risk of epidemics may be reduced at high altitudes (Watts et al., 2017). Regarding ticks, in Europe, *I. ricinus* is found up to approximately 2,000 m of altitude, depending on countries (Medlock et al., 2013). For example, in the colder, northern part of Europe, the altitudinal limit of *I. ricinus* is approximately 500 m above sea level in western Norway (Jore et al., 2011), 600 m in northeastern Scotland (Gilbert, 2010), rising to 1,100–1,500 m in northern Italy, Switzerland, and the Czech Republic (Rizzoli et al., 2002; Daniel et al., 2005; Burri et al., 2007), and could reach 2,000 m in Spain (Medlock et al., 2013). These limits revealed that favorable living conditions of *I. ricinus* depends on the region, and the absence of vectors necessarily breaks the pathogen transmission cycle despite the presence of hosts. The absence of the vector thus rhymes with a very low epidemic risk.

Ultimately, all environmental factors interact among them and with each partner of the vectorial triad. It is far from easy to understand such ecological complexity and reproduce their effects under controlled laboratory conditions. In addition, as all biological systems, the vectorial system evolves with species, which must adapt and evolve for survival. For example, chemical insecticides are widely used to control mosquitoes and ticks, which therefore have developed several mechanisms to counteract insecticide lethal effects, leading to maintaining pathogen transmission in resistant arthropod strains (Dusfour et al., 2019).

## Discussion

**FIGURE 4:**
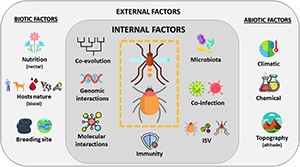
Overview of factors influencing the vectorial system. Vector capacity results from complex interactions of multiple factors influencing pathogen transmission by a vector. Internal factors like genetic, evolution, immunity, or interactions with other microorganisms are modulated by external parameters such as abiotic (like climate or topography) and biotic (like nutrition or hosts) factors (Lefevre et al., 2013). Created with Flaticon.com.

The vectorial capacity is a multifactorial process that includes several parameters influencing pathogen transmission, which, in mosquitoes and ticks, results from intensive and long-term interactions between vectors and their vertebrate hosts (Figure 4). Transmission is more an exception than a rule, and the majority of blood-circulating pathogens in vertebrates is not transmitted by vectors. Long-term coevolution of pathogens and vectors allowed finding the most appropriate evolutionary combination. Vector genome contains NIRVS, which are known to likely modulate pathogen transmission. In addition to vector genetics, the vector immune responses also condition the success of pathogen transmission. Vectors can naturally limit viral infection by deploying an arsenal of immune pathways (Toll, IMD, JAK/Stat, and RNAi pathways), each having an efficiency depending on the virus–vector combination. Moreover, the vector microbiome also modulates the vector competence; bacterial flora and ISV act on viral transmission. These factors, common for mosquitoes and ticks, differentially affect these two vectors due to their biology. In mosquitoes, the aquatic developmental phase (involving the immature stages: larvae and nymphs) influences the microbiota composition, which is critical to determine the vectorial capacity. Indeed, chemical and organic composition of mosquito larval breeding site also drastically impact the adult physiology, including pathogen transmission. As ticks are hemimetabolous arthropods (with incomplete metamorphosis), they are less sensitive to habitat variations than mosquitoes, thus influencing less their development. Particularities of ticks in blood feeding, blood digestion, and molting might explain to date the smaller number of arboviruses transmitted by ticks compared to mosquitoes. Contrary to mosquito-borne viruses, tick-borne viruses do not need to induce a high viremia in their vertebrate hosts to ensure vector infection, which is counterbalanced by a long blood meal lasting for hours to weeks and a longer tick life span (measured in years rather than in weeks or months for mosquitoes). Interestingly, it has been suggested that low viremia in hosts has contributed to favor non-viremic transmission between cofeeding ticks, which promotes the persistence of TBV in nature. Both mosquitoes and ticks are sensitive to temperature, hygrometry, and photoperiod, but tick movements are more dictated by vertebrate host movements than for mosquitoes: ticks passively wait for hosts (except for *Hyalomma*), while mosquitoes undertake active search for feeding. Some mosquito species are exclusively anthropophilic (*Ae. ae. aegypti* and *An. gambiae* complex), while for ticks, humans are more an accidental host, since no tick species are strictly anthropophilic. Consequently, since ticks are less associated with the human environment, they are less detrimental for human health than mosquitoes. However, in temperate regions like Europe, ticks remain the most important vectors; *I. ricinus* is the most common tick species in Europe and is also a vector of Lyme disease agent (Semenza and Suk, 2018).

Globally, mosquito spreading is favored by transportation and touristic activities that led to a large repartition of mosquitoes in the world and also that of their associated pathogens including mosquito-borne viruses. This wide distribution is due to the resistance to desiccation of mosquito eggs, allowing long trips in duration and distance as known for *Ae. albopictus* from Asia to America and Europe or for *Ae. ae. aegypti*, from Africa to America. Occasionally, ticks such as *Ixodes uriae* and *Ornithodoros maritimus* can move over long distances hooked on birds, but globally, hard tick’s dispersion is rather terrestrial *via* animals.

To date, our knowledge on arboviruses transmitted by mosquitoes and ticks showed that those with human importance are mainly transmitted by mosquitoes rather than by ticks. Tick-borne viruses are not transmitted by mosquitoes, and mosquito-borne viruses are rarely transmitted by ticks, suggesting important vector specificity in the arboviral transmission. Successful arbovirus transmission is conditioned by viral adaptation to vector physiology and behavior. Improving knowledge on virus–vector interactions, more advanced for mosquitoes than for ticks, will help in providing more reliable predictions of arboviruses emergence and implementing adapted vector control measures using, for example, *Wolbachia* artificially enriched mosquitoes, or genetically manipulated mosquitoes to boost their immune responses that ultimately may reduce their capacity to transmit pathogens.

## Author Contributions

All authors listed have made a substantial, direct, and intellectual contribution to the work, and approved it for publication.

## Conflict of Interest

The authors declare that the research was conducted in the absence of any commercial or financial relationships that could be construed as a potential conflict of interest.

## Publisher’s Note

All claims expressed in this article are solely those of the authors and do not necessarily represent those of their affiliated organizations, or those of the publisher, the editors and the reviewers. Any product that may be evaluated in this article, or claim that may be made by its manufacturer, is not guaranteed or endorsed by the publisher.
